# Polymorphisms in *CLDN1* are associated with age and differentiation of triple-negative breast cancer patients

**DOI:** 10.1042/BSR20181952

**Published:** 2019-04-23

**Authors:** Aimin Hu, Junyu Li, Shufang Ruan, Ying Fan, Yuqian Liao

**Affiliations:** 1 Department of Medical Oncology, Jiangxi Cancer Hospital, Nanchang, Jiangxi Province, P.R. China; 2 Department of Radiation Oncology, Jiangxi Cancer Hospital, Nanchang, Jiangxi province, P.R. China; 3 Department of Medical Oncology, Cancer institute and hospital, Peking Union Medical college, Chinese Academy of Medical science, Beijing, P.R. China

**Keywords:** Claudin, clinicopathological characteristics, polymorphism, TNBC

## Abstract

**Purpose:** Triple-negative breast cancer (TNBC) is a highly heterogeneous disease. It is very important to explore novel biomarkers to better clarify the characteristics of TNBC. It has been reported that polymorphisms in *claudin 1 (CLDN1)* are associated with risk of several cancers. But till now, there is no report about these polymorphisms and TNBC. **Patients and methods:** Between January 2004 and December 2013, 267 patients with stage I–III primary TNBC were included in our study. We investigated the association between polymorphisms in *CLDN1* gene and clinicopathological characteristics or survival of these patients. We used Haploview 4.2 software to identify Tag single nucleotide polymorphisms (SNPs). MassARRAY MALDI-TOF System was used for genotyping. **Results:** We found that rs10513846 GA genotype was associated with older age [*P*=0.013, hazard ratios (HR) = 2.231, 95% confidence interval (CI): 1.186–4.195]. Rs10513846 AA genotype carriers were more likely to develop grade 3 tumors (*P*=0.005, HR = 2.889, 95% CI: 1.389–6.007). And rs9283658 genotypes were also related to grade, more patients with grade 3 tumors were rs9283658 CC genotype carriers (*P*=0.023, HR = 0.446, 95% CI: 0.222–0.894). There was no association between polymorphisms in *CLDN1* and survival of TNBC patients. After multivariate analysis, tumor size (*P*=0.021, HR = 3.146, 95% CI: 1.185–8.354) and lymph node status (*P*<0.001, HR = 10.930, 95% CI: 3.276–36.470) were demonstrated to be independent prognostic factors. **Conclusion:** We first demonstrated that polymorphisms in *CLDN1* gene were associated with age and differentiation of TNBC patients.

## Introduction

Breast cancer is one of the most common cancers in women around the world [1]. It is a highly heterogeneous disease which for decades has been divided into several subgroups according to immunohistochemical staining (IHC) of three receptors: estrogen receptor (ER), progesterone receptor (PR), and epidermal growth factor receptor 2 (HER2) [2]. Triple-negative breast cancer (TNBC) is defined as lacking expression of ER, PR, and HER2. It accounts for 15–20% of all breast cancers and is characterized by enhanced invasiveness and metastatic capacity, young age of onset and poor prognosis [3]. However, even within TNBC patients, distinct response to treatments and prognosis was observed [4]. With the development of molecular profile, four to six distinct subtypes have been defined within TNBC, such as basal-like and claudin-low [5]. Investigators are making more efforts to explore novel biomarkers to clarify the characteristics of TNBC [6].

Claudins (CLDNs) are key cell adhesion molecules, which compose tight junctions (TJs), regulate paracellular permeability, and maintain cell polarity [7]. There are 27 members in CLDNs family, each member is predicted to possess four transmembrane domains with intracellular amino and carboxyl-termini in the cytoplasm and two extracellular loops [8]. It has been reported that CLDN1 expression levels were decreased in breast cancer [9], colorectal carcinoma [10], glioblastoma [11], and melanoma brain metastasis [12]. In contrast, only a few literatures reported about polymorphisms in *CLDN* genes and their role in cancer development [13,14]. Our study was designed to explore the relationship between genetic variants in *CLDN1* and clinicopathological characteristics or survival of TNBC.

## Materials and methods

### Study population

Between January 2004 and December 2013, 267 patients with stage I–III primary TNBC according to American Join Committee on Cancer 2010 classification [15] were included in our study. ER, PR, and HER2 status were evaluated according to the guidelines issued by the American Society of Clinical Oncology (ASCO) and the College of American Pathologists (CAP) in 2010 [16,17]. Tumors negative for ER, PR, and HER2 were defined as TNBCs. Clinical data such as age, tumor sizes, regional lymph node status, histopathologic grading, and vascular invasion were collected. Follow-up visits were performed every 3 months for 2 years, then every 6 months for 3 years, then annually. Patients were followed until December 2017 to collect data on recurrence and death.

This investigation was approved by the Institutional Review Board of the Chinese Academy of Medical Sciences Cancer Hospital and Jiangxi Cancer Hospital. It was conducted in accordance with the ethical standards of the Declaration of Helsinki and following the national and international guidelines. Written informed consent was obtained from all patients.

### Single nucleotide polymorphism selection and genotyping

Peripheral blood samples (5 ml) were collected from each patient upon recruitment and stored in −20°C for DNA extraction. Genotype data from *CLDN1* gene regions encompassing 5 kb of upstream and 5 kb of downstream flanking sequences were extracted from the HapMap Chinese Han population. Haploview 4.2 software was used to identify Tag single nucleotide polymorphisms (SNPs). The inclusion criteria were SNPs known in ethnic Han Chinese population and with a minor allele frequency (MAF) >0.05 and r^2^ > 0.8. A total of five candidate SNPs were selected for genotyping (Table 1). Primers and probes were designed by MassARRAY Typer 4.0 software. MassARRAY MALDI-TOF System (Sequenom Inc., San Diego, CA, U.S.A.) [18,19] was used for genotyping by the method described in the Sequenom Genotyping Protocol.

**Table 1:** Information for the SNPs genotyped in the present study

SNPs	Position	Location	Alleles	MAF
rs10513846	3:190313200	Intron variant	A/G	0.2017
rs1155884	3:190323165	Upstream variant 2KB	A/C	0.4631
rs8298	3:190305763	UTR variant 3 prime	C/T	0.2788
rs9842214	3:190305586	Intron variant	C/T	0.3223
rs9283658	3:190306476	Variant 3 prime	C/T	0.1749

### Statistical analyses

Statistical analysis was performed using SPSS version 18.0 (SPSS Inc, Chicago, IL, U.S.A.). The distribution of genotypes in patients with different clinicopathological characteristics was compared by two-sided Pearson’s χ^2^ tests, odds ratios (ORs) and 95% confidence intervals (CI) were calculated by logistic regression. Disease-free survival (DFS) was calculated from date of diagnosis to date of first locoregional recurrence, first distant metastasis, or death from any cause (whichever came first). Overall survival (OS) was calculated from date of diagnosis to date of death from any reason or last follow-up. Kaplan–Meier curves with the log-rank test were applied to estimate and compare 5-year DFS and OS rates of patients with different genotypes. Hazard ratios (HR) of recurrence/metastasis and death with 95% CI were estimated by Cox-regression model. The multivariate analysis was adjusted for age, histological grade, tumor size, lymph node status, and vascular invasion. All statistical tests were two-sided, and *P*<0.05 was considered significant.

## Results

### Clinical characteristics and survival of TNBC patients

A total of 267 TNBC patients were enrolled in our study. The median age at diagnosis is 47 years old (range from 23 to 75). The 5-year OS and DFS rate were 86.6 and 72.1%, respectively. More patients were ≤50 years old and with grade 3 tumors. 81 (30.3%), 135 (50.6%), and 51 (19.1%) subjects were diagnosed at stage I, II, and III. The association between clinicopathological characteristics and survival was listed in Table 2. Patients with grade 3 tumors had a significantly poorer 5-year OS rate than those with grade 1–2 tumors (78.3 vs 93.9%, *P*=0.029, HR = 2.445, 95% CI: 1.094–5.463). Tumor size and lymph node status were significantly associated with both DFS and OS. There was no significant association between age, vascular invasion, and TNBC survival. After multivariate analysis, tumor size (*P*=0.021, HR = 3.146, 95% CI: 1.185–8.354) and lymph node status (*P*<0.001, HR = 10.930, 95% CI: 3.276–36.470) were demonstrated to be independent prognostic factors.

**Table 2:** Clinicopathological characteristics and survival of TNBC

Variables	Patients (%)	5-year DFS (%)	HR (95% CI)	*P*-value	5-year OS (%)	HR (95% CI)	*P*-value
**Age**							
≤50	164 (61.4)	68.2	1 (Ref)		86.7	1 (Ref)	
> 50	103 (39.4)	78.7	0.590 (0.342–1.017)	0.058	86.4	0.876 (0.403–1.901)	0.737
**Grade**							
1–2	123 (46.1)	69.9	1 (Ref)		93.9	1 (Ref)	
3	144 (53.9)	76.0	1.112 (0.679–1.821)	0.674	78.3	2.445 (1.094–5.463)	0.029
**Vascular invasion**							
Negative	250 (93.6)	72.9	1 (Ref)		86.8	1 (Ref)	
Positive	17 (6.4)	60.7	1.603 (0.691–3.723)	0.272	80.4	2.261 (0.781–6.546)	0.133
**Tumor size**							
≤2cm	123 (46.1)	83.9	1 (Ref)		95.4	1 (Ref)	
>2cm	144 (53.9)	62.6	2.508 (1.440–4.369)	0.001	79.9	3.876 (1.473–10.198)	0.006
**Lymph node**							
Negative	159 (59.6)	80.7	1 (Ref)		98.0	1 (Ref)	
Positive	108 (40.4)	59.5	3.074 (1.843–5.126)	<0.001	71.3	13.252 (4.000–43.901)	<0.001
**TNM**							
I	81 (30.3)	86.3	1 (Ref)		100.0	1 (Ref)	
II	135 (50.6)	74.3	2.049 (0.933–4.499)	0.074	92.7	5.596 (0.716–43.745)	0.101
III	51 (19.1)	44.7	7.780 (3.534–17.127)	<0.001	52.0	34.557 (4.589–260.235)	0.001

**Abbreviations:** Ref, reference.

### Polymorphisms in *CLDN1* and clinicopathological features

The interactions between *CLDN1* genotypes and various clinicopathological characteristics were summarized in Supplementary Table 1. The distribution of rs10513846 genotypes was significantly associated with age and grade (Table 3). Rs10513846 GA genotype was associated with older age (*P*=0.013, HR = 2.231, 95% CI: 1.186–4.195). Rs10513846 AA genotype (*P*=0.005, HR = 2.889, 95% CI: 1.389–6.007) carriers were more likely to develop grade 3 tumors. And rs9283658 genotypes were also related to grade, more patients with grade 3 tumors were rs9283658 CC genotype carriers (*P*=0.023, HR = 0.446, 95% CI: 0.222–0.894). There was no significant association between other genetic variants in *CLDN1* and clinicopathological features.

**Table 3:** Relationship between genotypes and clinicopathological features

Variables	Age				Grade			
	≤50 (n, %)	>50 (n, %)	*P*-value	HR (95% CI)	1–2 (n, %)	3 (n, %)	*P*-value	HR (95% CI)
**rs10513846**								
**GG**	52 (31.7)	18 (17.5)		1 (Ref)	40 (32.5)	30 (20.8)		1 (Ref)
**GA**	79 (48.2)	61 (59.2)	0.013	2.231 (1.186–4.195)	65 (52.8)	75 (52.1)	0.144	1.538 (0.863–2.743)
**AA**	33 (20.1)	24 (23.3)	0.053	2.101 (0.992–4.452)	18 (14.7)	39 (27.1)	0.005	2.889 (1.389–6.007)
**rs9283658**								
**CC**	40 (24.4)	31 (30.1)		1 (Ref)	26 (21.1)	45 (31.3)		1 (Ref)
**CT**	79 (48.2)	55 (53.4)	0.718	0.898 (0.502–1.607)	62 (50.4)	72 (50.0)	0.185	0.671 (0.372–1.211)
**TT**	45 (27.4)	17 (16.5)	0.053	0.487 (0.235–1.010)	35 (28.5)	27 (18.7)	0.023	0.446 (0.222–0.894)

**Abbreviations:** n, number of patient; Ref, reference.

### Polymorphisms in *CLDN1* and survival of TNBC patients

Tables 4 and 5 listed the 5-year DFS and OS rate for patients with different genotypes. There was no association between polymorphisms in *CLDN1* and survival of TNBC patients. Rs9842214 TT genotype carriers had less DFS rate than CC genotype carriers, the 5-year DFS was 74.8 and 39.8%, respectively. However, the difference was not significant, *P*-values were 0.050 and 0.056 in univariant and multi-variant analyses, respectively. Since only ten patients carried rs9842214 TT genotype, the results need to be verified.

**Table 4:** *CLDN1* genotypes and DFS

Variables	Patients (%)	5-year DFS(%)	Crude		Adjusted	
			HR (95% CI)	*P*-value	HR (95% CI)	*P*-value
**rs10513846**						
**GG**	70 (26.2)	69.6	1 (Ref)		1 (Ref)	
**GA**	140 (52.4)	69.7	0.961 (0.544–1.698)	0.891	0.933 (0.520–1.675)	0.817
**AA**	57 (21.4)	82.3	0.763 (0.360–1.617)	0.480	0.757 (0.349–1.640)	0.480
**rs1155884**						
**AA**	144 (54.0)	78.0	1 (Ref)		1 (Ref)	
**AC**	98 (36.7)	62.2	1.470 (0.882–2.449)	0.139	1.278 (0.756–2.161)	0.359
**CC**	25 (9.3)	78.2	1.032 (0.399–2.667)	0.949	1.009 (0.382–2.669)	0.986
**rs8298**						
**CC**	157 (58.8)	74.3	1 (Ref)		1 (Ref)	
**CT**	83 (31.1)	69.8	1.006 (0.577–1.754)	0.983	1.068 (0.608–1.876)	0.819
**TT**	27 (10.1)	63.2	1.570 (0.756–3.263)	0.227	1.656 (0.783–3.500)	0.187
**rs9842214**						
**CC**	167 (62.5)	74.8	1 (Ref)		1 (Ref)	
**CT**	90 (33.7)	68.2	1.003 (0.585–1.721)	0.991	1.166 (0.674–2.017)	0.583
**TT**	10 (3.8)	38.9	2.542 (0.999–6.470)	0.050	2.527 (0.976–6.543)	0.056
**rs9283658**						
**CC**	71 (26.6)	83.7	1 (Ref)		1 (Ref)	
**CT**	134 (50.2)	68.0	1.445 (0.750–2.787)	0.272	1.436 (0.742–2.781)	0.283
**TT**	62 (23.2)	71.7	1.571 (0.749–3.295)	0.232	1.575 (0.736–3.369)	0.242

**Abbreviation:** Ref, reference.

**Table 5:** *CLDN1* genotypes and OS

Variables	5-year OS (%)	Crude		Adjusted	
		HR (95% CI)	*P*-value	HR (95% CI)	*P*-value
**rs10513846**					
**GG**	91.4	1 (Ref)		1 (Ref)	
**GA**	86.7	2.222 (0.747–6.606)	0.151	1.514 (0.491–4.673)	0.471
**AA**	81.1	2.561 (0.747–8.779)	0.135	1.803 (0.492–6.615)	0.374
**rs1155884**					
**AA**	88.8	1 (Ref)		1 (Ref)	
**AC**	82.9	1.984 (0.911–4.321)	0.085	1.856 (0.833–4.135)	0.130
**CC**	89.7	1.144 (0.253–5.171)	0.861	1.121 (0.235–5.339)	0.886
**rs8298**					
**CC**	89.2	1 (Ref)		1 (Ref)	
**CT**	82.9	1.256 (0.564–2.798)	0.577	1.169 (0.504–2.716)	0.716
**TT**	86.0	0.995 (0.287–3.445)	0.994	1.266 (0.347–4.616)	0.721
**rs9842214**					
**CC**	87.9	1 (Ref)		1 (Ref)	
**CT**	86.3	1.058 (0.471–2.380)	0.891	1.419 (0.613–3.285)	0.413
**TT**	75.0	1.587 (0.364–6.922)	0.538	2.510 (0.549–11.473)	0.235
**rs9283658**					
**CC**	81.6	1 (Ref)		1 (Ref)	
**CT**	88.4	0.769 (0.318–1.860)	0.560	0.782 (0.330–1.855)	0.577
**TT**	87.9	0.807 (0.246–2.653)	0.724	0.573 (0.187–1.760)	0.331

**Abbreviation:** Ref, reference.

## Discussion

The principle functions of TJs include preventing the mixing of membrane proteins between the apical and basolateral membranes; and controlling the paracellular passage of ions and solutes in between cells [20]. TJs play important roles in tumor progression and metastasis [21]. A disruption of TJs during tumorigenesis generally leads to invasiveness, loss of cohesion, and lack of differentiation in cancer cells [21]. CLDNs are the first components identified to be involved in sealing in TJs [22]. To date, 27 CLDN family members have been identified; each CLDN has a different tissue-expression pattern and function [22,23]. Thus, CLDNs may serve both as biomarkers in detecting cancer and as possible targets in cancer therapeutics [24]. CLDN1 is one of the most commonly investigated CLDNs, but the association between polymorphisms in *CLDN1* and TNBC has never been reported.

*CLDN1* is a 17 kb gene that codes for a 3.4 kb transcript which translates to an important protein CLDN1 [25]. It has been reported that polymorphisms in *CLDN1* are associated with the risk of cancer [14], small vessel vascular dementia [26], leukoaraiosis [27], and hepatitis C virus infection [28,29]. In Hahn-Strömberg V’s study, they found that *CLDN1* rs9869263 genotype was related to risk of colon cancer and polymorphisms in *CLDN7* were associated with differentiation and age of colon cancer [14]. Chen et al. reported that *CLDN1* rs17501976 polymorphism was significantly associated with a decreased susceptibility to colorectal cancer in a Chinese population [30]. Polymorphisms investigated in our study have never been reported in cancer patients. We first demonstrated that rs10513846 and rs9283658 genotypes were significantly associated with age and grade in TNBC patients. As age and differentiation have been proved to be prognostic factors for breast cancer [31,32], our results indicate the potential role of polymorphisms in *CLDN1* as biomarkers for tumor invasion or prognosis.

Though researches about polymorphisms in *CLDN1* are rare, protein CLDN1 has been widely investigated in cancers. CLDN1 can promote or suppress tumor proliferation in different cancers or even in different histological subtypes of the same cancer. The over expression of CLDN1 has been reported to increase cell invasion in colorectal cancer [33] and oral squamous cell carcinoma (OSCC) [34]. CLDN1 has long been considered as a tumor suppressor in breast cancer. But recently, some studies showed that the expression level of CLDN1 was low in luminal-like and claudin-low breast cancers, while the expression level of CLDN1 was high in basal-like, most ER negative, BRCA1, medullary breast cancers [35]. Whether CLDN1 plays tumor-facilitating role in basal-like breast cancer or TNBC still needs to be proved.

**Figure 1:**
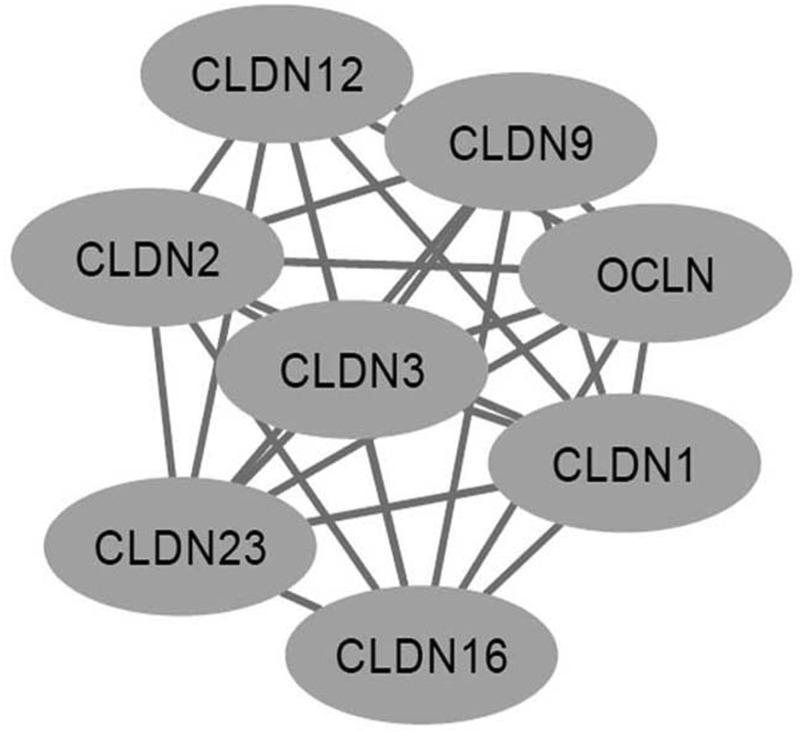
Interactions between CLDN1 and related proteins

Down-regulation of CLDN1 was associated with shorter DFS of breast cancer patients [9]. In TNBC, CLDN1-negative phenotype also predicted poor prognosis [36,37]. In our study, no association between polymorphisms in *CLDN1* and survival of TNBC patients was observed. There are some explanations. First, the sample size was relatively small and we did not do subgroup analysis. Second, though polymorphisms in *CLDN1* might influence the expression level of CLDN1 protein, the complex interactions between CLDN family members could also affect the results. By using STRING, we found that CLDN1 interacts with some other proteins in CLDN family, such as CLDN2, CLDN3, CLDN6, CLDN12 and so on (Figure 1). CLDN 12 expression could be clinically useful for predicting the survival of the ER-negative subgroup of patients with breast cancer [38]. High expression of CLDN6 confers chemoresistance on breast cancer [39]. So, combination analysis of CLDN1 and other related proteins might help us to better understand the role of CLDN1 in TNBC.

The expression level of CLDN1 was found to be associated with tumor differentiation and age in several kinds of cancers. In OSCCs, the highest expression of CLDN1 was observed in well-differentiated OSCCs, whereas poorly differentiated OSCCs exhibited mostly negative staining for CLDN1 [40]. In hepatocellular cancer, down-regulation of CLDN1 was associated with poor differentiation [41]. In basal-like breast cancer patients, the highest level of CLDN1 protein expression was observed in patients who were older than 55 years of age [42]. So, we assume that polymorphisms in *CLDN1* might influence the expression level of CLDN1 protein and then influence the tumor differentiation. However, the underlying mechanisms need to be further investigated. Our results also suggested that polymorphisms in *CLDN1* might help us to identify subtypes of TNBC, as TNBC patients with different age and grade were proved to have unique molecular features [32,43].

## Conclusion

In conclusion, we first demonstrated that polymorphisms in *CLDN1* gene were associated with age and differentiation of TNBC patients. Since most polymorphisms have never been reported and the underlying mechanisms are still unknown, more researches are needed to verify our results.

## Supporting information

**Supplemental Table S1:** Relationship between genotypes and clinicopathological features

## Abbreviations

BRCA: breast cancer susceptibility gene
CI: confidence interval
CLDN 1: *Claudin* 1
DFS: disease-free survival
ER: estrogen receptor
HER2: epidermal growth factor receptor 2
HR: hazard ratio
OS: overall survival
OSCC: oral squamous cell carcinoma
PR: progesterone receptor
SNP: single nucleotide polymorphism
TJ: tight junction
TNBC: triple-negative breast cancer
