# Supplementary material for: Polymorphisms in CLDN1 are associated with age and differentiation of triple-negative breast cancer patients
Source: Biosci Rep. 2019 Apr 23;39(4):BSR20181952. doi: 10.1042/BSR20181952 (PMC6481238; doi:10.1042/BSR20181952)
Supplement: Supplementary file 1 [file bsr20181952_Supp1.pdf]

**Supplementary Table 1. Relationship between genotypes and clinicopathological features**

| Variables | Age | | | Grade | | | Tumor size | | | Lymph node status | | | Vascular invasion | | |
|---|---|---|---|---|---|---|---|---|---|---|---|---|---|---|---|
| | ≤50 | >50 | *P* | 1-2 | 3 | *P* | ≤2cm | >2cm | *P* | negative | positive | *P* | negative | positive | *P* |
| **rs10513846** | | | | | | | | | | | | | | | |
| **GG** | 52 | 18 | 1(Ref) | 40 | 30 | 1(Ref) | 35 | 35 | 1(Ref) | 44 | 26 | 1(Ref) | 66 | 4 | 1(Ref) |
| **GA** | 79 | 61 | 0.013 | 65 | 75 | 0.144 | 68 | 72 | 0.845 | 80 | 60 | 0.428 | 130 | 10 | 0.696 |
| **AA** | 33 | 24 | 0.053 | 18 | 39 | 0.005 | 20 | 37 | 0.093 | 35 | 22 | 0.867 | 54 | 3 | 0.912 |
| **rs1155884** | | | | | | | | | | | | | | | |
| **AA** | 91 | 53 | 1(Ref) | 67 | 77 | 1(Ref) | 65 | 79 | 1(Ref) | 94 | 50 | 1(Ref) | 137 | 7 | 1(Ref) |
| **AC** | 55 | 43 | 0.270 | 46 | 52 | 0.950 | 46 | 52 | 0.783 | 52 | 46 | 0.057 | 90 | 8 | 0.301 |
| **CC** | 18 | 7 | 0.398 | 10 | 15 | 0.546 | 12 | 13 | 0.791 | 13 | 12 | 0.207 | 23 | 2 | 0.523 |
| **rs8298** | | | | | | | | | | | | | | | |
| **CC** | 99 | 58 | 1(Ref) | 71 | 86 | 1(Ref) | 69 | 88 | 1(Ref) | 95 | 62 | 1(Ref) | 148 | 9 | 1(Ref) |
| **CT** | 48 | 35 | 0.430 | 39 | 44 | 0.794 | 41 | 42 | 0.421 | 48 | 35 | 0.688 | 77 | 6 | 0.649 |
| **TT** | 17 | 10 | 0.993 | 13 | 14 | 0.778 | 13 | 14 | 0.685 | 16 | 11 | 0.902 | 25 | 2 | 0.735 |
| **rs9842214** | | | | | | | | | | | | | | | |
| **CC** | 104 | 63 | 1(Ref) | 75 | 92 | 1(Ref) | 72 | 95 | 1(Ref) | 100 | 67 | 1(Ref) | 156 | 11 | 1(Ref) |
| **CT** | 53 | 37 | 0.595 | 45 | 45 | 0.436 | 48 | 42 | 0.118 | 53 | 37 | 0.877 | 85 | 5 | 0.744 |
| **TT** | 7 | 3 | 0.625 | 3 | 7 | 0.363 | 3 | 7 | 0.420 | 6 | 4 | 0.994 | 9 | 1 | 0.679 |
| **rs9283658** | | | | | | | | | | | | | | | |
| **CC** | 40 | 31 | 1(Ref) | 26 | 45 | 1(Ref) | 29 | 42 | 1(Ref) | 41 | 30 | 1(Ref) | 67 | 4 | 1(Ref) |
| **CT** | 79 | 55 | 0.718 | 62 | 72 | 0.185 | 63 | 71 | 0.398 | 80 | 54 | 0.787 | 125 | 9 | 0.762 |
| **TT** | 45 | 17 | 0.053 | 35 | 27 | 0.023 | 31 | 31 | 0.291 | 38 | 24 | 0.678 | 58 | 4 | 0.843 |
